# Rapid and Reliable Detection of SARS-CoV-2 Using Direct RT-LAMP

**DOI:** 10.3390/diagnostics12040828

**Published:** 2022-03-28

**Authors:** Sherif A. El-Kafrawy, Mai M. El-Daly, Ahmed M. Hassan, Steve M. Harakeh, Thamir A. Alandijany, Esam I. Azhar

**Affiliations:** 1 Special Infectious Agents Unit-BSL3, King Fahd Medical Research Center, King Abdulaziz University, Jeddah 21589, Saudi Arabia; saelkfrawy@kau.edu.sa (S.A.E.-K.); meldaly@kau.edu.sa (M.M.E.-D.); hmsahmed@kau.edu.sa (A.M.H.); sharakeh@kau.edu.sa (S.M.H.); talandijany@kau.edu.sa (T.A.A.); 2 Department of Medical Laboratory Sciences, Faculty of Applied Medical Sciences, King Abdulaziz University, Jeddah 21589, Saudi Arabia

**Keywords:** RT-LAMP, direct assay, SARS-CoV-2, COVID-19, molecular assay, PCR

## Abstract

**Background:** The global pandemic coronavirus SARS-CoV-2 has a healthcare, social and economic burden. To limit the spread of the virus, the World Health Organization (WHO) urgently called for extensive screening of suspected individuals; thus, a quick, simple, and sensitive diagnostic assay is always in need. **Methods:** We applied reverse transcription-loop-mediated isothermal amplification (RT-LAMP) for the detection of SARS-CoV-2. The RT-LAMP method was optimized by evaluating two fluorescence amplification mixes and several reaction times, and results were compared to the standard real-time RT-PCR (rtRT-PCR). The assay was validated using 200 nasopharyngeal swabs collected in viral transport media (62 positive for SARS-CoV-2, and 138 negative for SARS-CoV-2 detected by the rtRT-PCR method). The samples were diluted 1:4 in diethylpyrocarbonate (DEPC)-treated water, utilized for RT-LAMP using different singleplex and multiplex sets of LAMP primers (N gene, S gene, and orf1ab gene), and incubated at 65 °C using real-time PCR 7500. **Results:** Our direct detection with the RT-LAMP protocol showed 100% concordance (sensitivity and specificity) with the standard protocol used for the detection of SARS-CoV-2 nucleic acid. **Conclusions:** In this study, we set up a rapid, simple, and sensitive RT-LAMP assay for the detection of SARS-CoV-2 in clinical samples. The assay is suitable for point of care detection in public hospitals, medical centers in rural areas, and in transportation hubs.

## 1. Introduction

Coronavirus Disease 2019 (COVID-19) is a widespread pandemic caused by the severe acute respiratory syndrome coronavirus 2 (SARS-CoV-2), resulting in a major worldwide health, social and economic problem. SARS-CoV-2 is the seventh known coronavirus that causes human diseases. It is an enveloped, non-segmented, single-stranded, positive-sense RNA virus similar to severe acute respiratory syndrome coronavirus (SARS-CoV) and Middle East respiratory syndrome coronavirus (MERS-CoV) [1] but SARS-CoV-2 infection has a stronger human-to-human transmission capacity [2,3].

The limited testing capacity during the COVID-19 pandemic resulted in only symptomatic people being tested for SARS-CoV-2 infection, while later studies have confirmed that several cases infected with SARS-CoV-2 are asymptomatic contagious carriers [4,5]. To address this challenge, a quick, simple, specific, and sensitive method would be highly required for rapid diagnosis of the virus to control the spread of SARS-CoV-2 in the populations. With such a test, the infected patients could be identified at an early stage and isolated to avoid the spread of the infection [6].

Testing of nucleic acid through viral RNA extraction and quantitative PCR (qRT-PCR) is the practical and standard method of testing with high specificity and sensitivity. The reagents, supplies, and instruments needed for sample extraction and assay performance might be scarce or overpriced in the time of the pandemic waves. Although qRT-PCR is a reliable and sensitive assay, it is time-consuming as it requires about 2 h for the assay in addition to about 90 min for the RNA extraction [6]. While PCR techniques require different working conditions in terms of temperatures during reaction steps, isothermal amplification assays such as Loop-mediated Isothermal Amplification (LAMP), Nucleic Acid Sequence-based Amplification (NASBA), Strand Displacement Amplification (SDA), Recombinase Polymerase Amplification (RPA), and Rolling Circle Amplification (RCA), work at a stable temperature throughout the reaction. Of the isothermal amplification techniques, LAMP has several advantages for nucleic acid amplification, including shorter amplification time, tolerance to inhibitory substances present in clinical specimens, and the high variety of commercially available reagents [7].

Reverse-Transcriptase Loop-mediated isothermal amplification (RT-LAMP) is an alternative to qRT-PCR. It is faster and requires fewer resources and is used for the amplification of RNA templates with strong strand displacement activity and tolerance for elevated temperatures [8]. The RT-LAMP method was applied to pathogen detection such as the MERS-CoV, Zika virus, West Nile virus, Ebola virus, yellow fever virus, and a variety of other pathogens [9,10,11,12,13,14,15,16,17,18,19,20].

The LAMP reaction takes place at a constant temperature, and the target DNA can be amplified within 30 min. The LAMP method employs four or six primers to bind six regions of a target DNA with high specificity [21,22]. Initially, the LAMP uses four primers for the detection of DNA [21,22]. Next, the stretching of two-loop primers could shorten half of the time required for the initial LAMP reaction [23]. The accessibility of RT-LAMP allows both reverse transcription and LAMP to be combined in a single reaction. The application of RT-LAMP for the amplification of SARS-CoV-2 is expected to reduce the reaction time.

In a continuation to our work to evaluate SARS-CoV-2 detection assays that can be used in economically restricted areas in the time when resources and supplies are scarce and unavailable [24], we report here the evaluation of a RT-LAMP assay for the rapid, sensitive and specific detection of SARS-CoV-2 in clinical samples. The assay was set up using fluorescent detection to facilitate the automatic call of the results to avoid the uncertainty of human error when using color-based RT-LAMP amplification mixes.

## 2. Methods

### 2.1. SARS-CoV-2 Samples

The study was designed to investigate the sensitivity and specificity of the RT-LAMP assay to assess its suitability as an alternative to the qRT-PCR assay for the detection of SARS-CoV-2 viral RNA in clinical specimens. The clinical samples used in this study were previously tested using the qRT-PCR assay.

This study also evaluated different primer sets targeting different viral genome regions as means to meet the WHO criteria for testing the viral genome in two different genetic regions. The study evaluated the use of clinical samples without RNA extraction as a means to omit the extraction step as an attempt to reduce time and cost of the assay. The performance of two RT-LAMP amplification mixes was also evaluated. This study was conducted at the Special Infectious Agents Unit (SIAU), King Fahd Medical Research Center, King Abdulaziz University, Jeddah, Saudi Arabia.

### 2.2. Viral RNA Extraction

SARS-CoV-2 viral isolate titration ranging from 3.16 × 10^5^ to 3.16 × 10^−2^ pfu/mL was performed by 10-fold serial dilution of a clinical isolate (SARS-CoV-2/human/SAU/85791C/2020, Genbank accession number MT630432.1) and the dilution series were either used for RNA extraction or for direct detection of the virus without RNA extraction. The serial dilution and processing were performed in the biosafety level 3 facility of the SIAU. Processing of nasopharyngeal samples was performed in a Class II biosafety cabinet in a negative pressure BSL-2 lab. The extraction was performed using the ExiPrep™ 96 Viral DNA/RNA kit (BiONEER, Seoul, South Korea) on the automatic extractor BiONEER according to the manufacturer’s instructions with a 210 µL sample volume and a 50 µL elution volume.

### 2.3. Reverse Transcription Real-Time PCR

The PowerChek 2019-nCov Real-Time PCR Kit (Seoul, Korea) was used for the detection of SARS-CoV-2, briefly, by adding 5 µL of the extracted RNA or 5 µL of the diluted sample (1:4) to the reaction master mix consisting of 11 µL RT-PCR Premix and 4 µL of Primer/Probe Mix for a total reaction volume of 20 µL. As per WHO recommendations, two targets were separately amplified: the E gene of beta coronaviruses and the RdRp gene of the SARS-CoV-2 viral genome [25]. Both probes were labeled with FAM. The reaction mixture included an internal control for PCR inhibition with a VIC-labeled probe. The reaction’s thermal profile was 50 °C for 30 min, then 95 °C for 10 min, followed by 40 cycles of 95 °C for 15 sec and 60 °C for 1 min on the LightCycler^®^ 480 Instrument II (Roche, Germany). The assay is marked for in vitro diagnostic use (IVD) and is approved by the authorities for case identification.

### 2.4. Sample Preparation and RT-LAMP

The RT-LAMP assay was first evaluated by comparing the RT-PCR results of the isolated serial dilution using 21 extracted samples. The serial dilutions of the SARS-CoV-2 clinical isolate and 200 nasopharyngeal swabs (62 positive for SARS-CoV-2, and 138 negative for SARS-CoV-2) collected from suspected COVID-19 cases in viral transport media (VTM) were diluted 1:4 in DEPC-treated water. The performance of the direct assay was evaluated by comparing the direct RT-LAMP assay for the dilution series with and without heating the samples prior to RT-LAMP amplification. Two sets of diluted samples were added in a 0.2 mL 96-well plate sealed with an adhesive film to minimize carryover contamination and ensure biosafety, one set was incubated at 95 °C for 5 min, followed by cooling to 4 °C for 5 min in a thermal cycler, and the other set was not heated at 95 °C prior RT-LAMP to reduce the overall time of the assay by 10 min.

### 2.5. Evaluation of the Singleplex and Multiplex RT-LAMP Assay

The RT-LAMP primer sets used in this study were targeting the open reading frame (ORF1ab), Spike (S) [26], and Nucleocapsid (N) gene [27]. Three tubes containing 10× primer mix working stock were prepared for each target gene and used in the RT-LAMP assay of each target either separately (singleplex) or in binary combination (multiplex).

The RT-LAMP assay was performed by preparing the amplification mix as 1× WarmStart^®^ LAMP Kit DNA & RNA (New England Biolabs, Ipswich, MA, USA), 0.5 µL of the 50× fluorescent dye with 2.5 µL of 10× LAMP primer mix (each), and the volume was completed to 20 µL with DEPC treated water, finally, 5.0 µL of the test sample (either extracted RNA, heated diluted sample or unheated diluted sample) were added to each respective tube to give a total reaction volume of 25 µL. Samples were incubated at 65 °C using real-time PCR 7500 (Applied BioSystems, Waltham, MA, USA) and fluorescence was collected via the FAM filter of the instrument. In order to reduce the assay time further, we compared the performance of the direct RT-LAMP assay at different incubation times of 60, 45, 40, 35, and 30 min at 65 °C.

### 2.6. Evaluation of the Assay Sensitivity

The diagnostic performance of the RT-LAMP assay was calculated by comparison with the results of the reference standard assay (qRT-PCR). Sensitivity = true positives/(true positives + false negatives).

Similarly, specificity was calculated to estimate the proportion of individuals who were unaffected by COVID-19 and remained negative in the RT-LAMP assay. Specificity = true negatives/(true negatives + false positives).

### 2.7. Limit of Detection (LOD)

The LOD of the assay was evaluated by testing a dilution series of the local SARS-CoV-2 clinical isolate with known viral titer. The 10-fold serial dilution samples (ranging from 3.16 × 10^5^ to 3.16 × 10^−2^ pfu/mL) were tested using the different combinations of primer sets (N-Gene/S-123, N-Gene/ORF1ab, ORF1ab/S-123, and N-Gene/S-123/ORF1ab) and the different reaction conditions (reaction times and sample dilutions). Samples were tested in triplicates, the LOD of the assays was identified as the last dilution which gave positive results in all triplicate samples of this dilution. Results were obtained from three independent experiments.

### 2.8. Cross-Reactivity with Other Coronaviruses

The cross-reactivity of the assay was evaluated by testing a panel of dilution series of MERS-CoV isolates (MERS-CoV/Hu/Taif/SA/2015) using the final RT-LAMP reaction conditions.

## 3. Results

**Figure 1:**
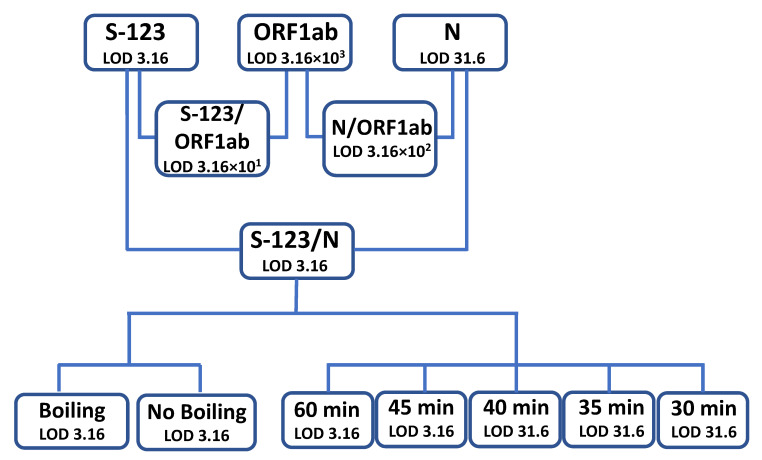
Flowchart demonstrating the limit of detection (LOD) for the methods used in the study including primer regions and sample treatment.

In this study, the RT-LAMP method was evaluated using live SARS-CoV-2 cell culture supernatant from a clinical isolate that was serially diluted (10-fold), ranging from 3.16 × 10^5^ to 3.16 × 10^−2^ pfu/mL. These dilutions were tested using RT-LAMP to detect the ORF1ab, N-Gene, and S-123. As a proof of concept, the isolate samples dilutions were analyzed using RNA extraction followed by rtRT-PCR and RT-LAMP using the three primer sets either separately or in combinations. The standard rtRT-PCR protocol served as a reference to evaluate the performance, sensitivity, and specificity of the RT-LAMP PCR protocol. Figure 1 shows a flowchart summarizing the workflow and the results generated in the study.

### 3.1. Evaluation of the Primer Sets

The S-123 primer set showed a LOD of 3.16 pfu/mL while the N-Gene primer set showed a LOD of 3.16 × 10^1^ pfu/mL. The ORF1ab primer set showed the highest LOD of 3.16 × 10^3^ pfu/mL. The combination of N-gene primers with the S-123 gene primers set was found to be the most sensitive when used in the multiplex assay with a LOD of 3.16 pfu/mL. The other two primers set combinations showed a LOD of 3.16 × 10^2^ pfu/mL for the ORF1ab/N-Gene primer mix and 3.16 × 10^1^ pfu/mL for the ORF1ab/S-123 primer mix. The assay was performed by incubation at 65 °C for 60 min as recommended by the manufacturer. The most sensitive primer set combination S-123/N-gene (LOD 3.16 pfu/mL) was then used for the evaluation of the assay. The assay did not show inter-run variability when tested on different testing days.

### 3.2. Evaluation of Specimen Boiling

In order to reduce the assay time, we evaluated omitting the boiling step where the sample is diluted with DEPC treated water (1:4) and heated at 95 °C followed by cooling at 4 °C. Omitting the boiling step showed the same LOD (3.16 pfu/mL) and resulted in 100% concordance with the boiling protocol. The comparison was performed using the viral isolates dilution series.

### 3.3. Evaluation of RT-LAMP Reaction Time

**Figure 2:**
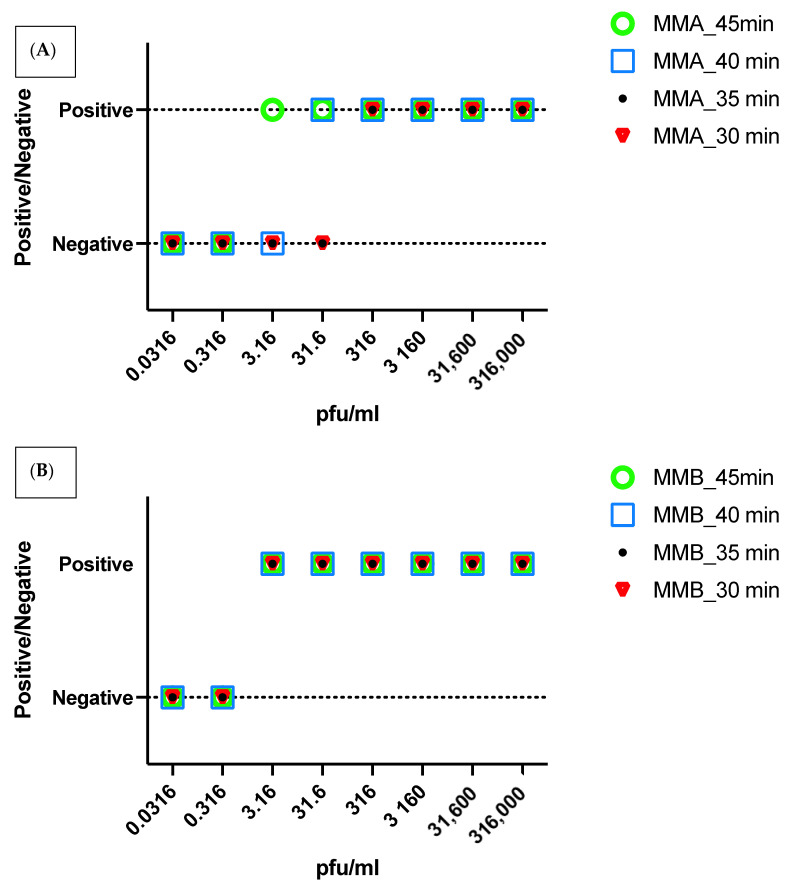
The LOD of MMix-A (**A**) and MMix-B (**B**) was evaluated at 60- 45- 40-, 35- and 30-min using cell culture isolates. The shortest time to give the highest level of detection (3.16 pfu/mL) for both assays was 45- and 30-min using MMix-A and MMix-B, respectively.

In order to reduce the assay time further, the performance of the assay was evaluated at different incubation times (60-, 45-, 40-, 35-, and 30-min). The LOD varied with reaction time with the reaction time of 45 min showing the same LOD as the recommended 60 min. The shorter reaction times of 30-, 35- and 40-min showed a higher limit of detection of 3.16 × 10^1^. This comparison was performed with the master mix A (MMA) (Figure 2A).

### 3.4. Evaluation of the Type of Mastermix

**Figure 3:**
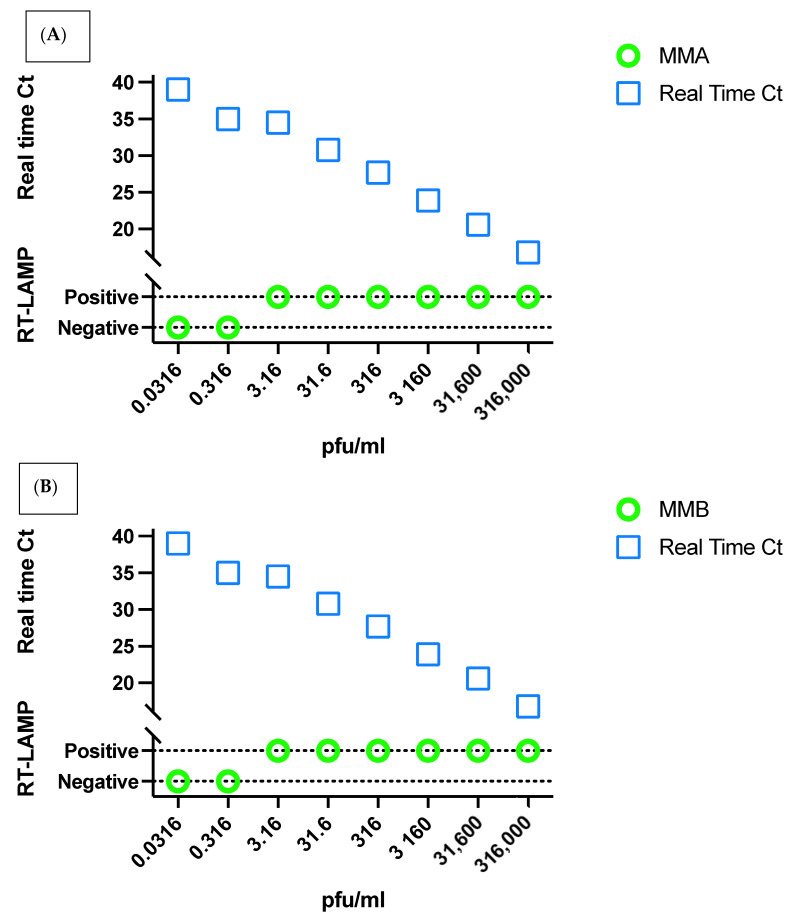
Comparison between RT-LAMP assay for MMix-A (**A**) and MMix-B (**B**) and the standard IVD approved SARS-CoV-2 diagnostic assay real-time RT-PCR. The lower part of the *Y*-axis represents the qualitative results of the RT-LAMP assay (positive/negative) while the upper part represents the corresponding Ct values when tested by real-time RT-PCR.

Two RT-Lamp amplification reagents from New England *Biolabs* were used (MMix-A and MMix-B). MMix-A is commercially available (cat# E1700L), while MMix-B is an experimental lyo-compatible reagent. The LOD of both reagents was evaluated at 60-, 45-, 40-, 35-, and 30-min using cell culture isolates (Figure 2). The shortest time giving the highest LOD for both assays was 45 min; where MMix-A showed LOD of 3.16 pfu/mL and MMix-B showed a LOD of 3.16 × 10^−1^ pfu/mL whereas MMix-B showed a LOD of 3.16 pfu/mL at 30 min reaction time. As the LOD of diagnostic rtRT-PCR was found to be 3.16 pfu/mL, we used a reaction time of 45 min for MMix-A and 30 min for MMix-B to match the LOD of the standard assay in the clinical evaluation of the assays as shown in Figure 3.

### 3.5. Validation of the Assay in Clinical Samples

**Figure 4:**
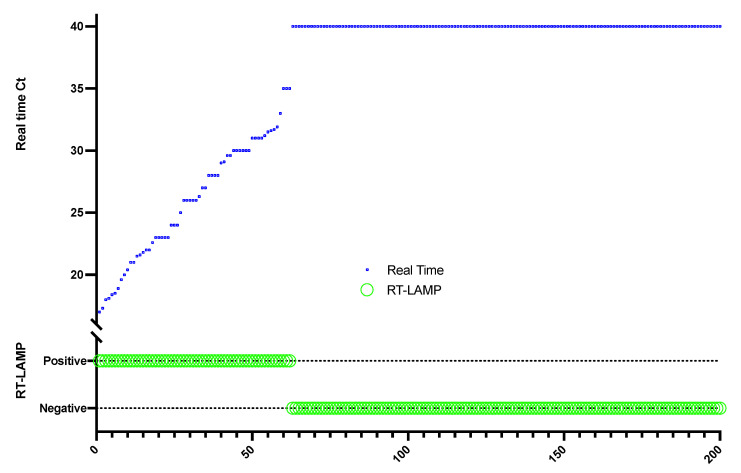
Clinical evaluation of the assay using respiratory specimens routinely sent for SARS-CoV-2 diagnosis (62 positive and 138 negative) by real-time RT-PCR. The positive specimens showed Ct values ranging from 16.8 to 35 in the real-time RT-PCR. The lower part of the *Y*-axis represents the qualitative results of the RT-LAMP assay (positive/negative) while the upper part represents the corresponding Ct values when tested by real-time RT-PCR.

The clinical evaluation of the samples was performed on 200 patient respiratory specimens that were sent to the SIAU for the laboratory diagnosis of SARS-CoV-2, of which 62 specimens were positive and 138 were negative by rtRT-PCR. The positive specimens showed Ct values ranging from 16.8 to 35 in the rtRT-PCR. The clinical samples showed 100% concordance when tested with the RT-LAMP assay compared to the standard rtRT-PCR used for diagnosis resulting in a sensitivity and specificity of 100% as shown in Figure 4. The evaluation RT-LAMP assay was performed using the primer combination S123/N gene and both the amplification mixes MMix-A with a reaction time of 45 min and MMix-B with a reaction time of 30 min directly without boiling. The clinical evaluation of the RT-LAMP assays was performed on the same day of the rtRT-PCR assay to retain the same sample conditions for both assays.

### 3.6. Cross-Reactivity with Other Coronaviruses

The cross-reactivity of the assay was evaluated by testing the primers used in this study on a dilution series of a MERS-CoV isolate. All the dilutions of the MERS-CoV isolate showed negative results while the different dilutions of the SARS-CoV-2 isolates showed positive results.

## 4. Discussion

In a perfect situation, a point of care test kit should be portable and does not need any complex instruments to be utilized in airports and train stations and near patient clinics, especially in regional hospitals and medical centers in rural areas (Huang, Lim et al. 2020). In this study, we report an efficient, sensitive, specific, and fluorescence-based RT-LAMP assay for the molecular detection of SARS-CoV-2 in clinical samples. The assay utilizes two sets of primers targeting two of the viral genome regions (S and N) as per the recommendations of the WHO. The assay used the direct approach for the detection of the virus without RNA extraction in order to reduce the cost, time, and dependence on the supply chain in times of scarcity. The assay condition of the RT-LAMP reaction helped to reduce the time of the reaction to 30 min.

RT-LAMP assay from this study was compared with the gold standard diagnostic assay real-time RT-PCR that is IVD approved for the diagnosis of SARS-CoV-2. As a proof of concept, the assay was first evaluated using RNA extracts of SARS-CoV-2 isolates with known viral titer. Next, the direct approach (without RNA extraction) was evaluated. In our previous study [24], the direct real-time RT-PCR showed the highest LOD using the boiling method of the clinical samples before submitting to the real-time RT-PCR reaction. In this study, we evaluated the three primer sets, S, N, and ORF1ab, by estimating the LOD of the assay with each primer set separately.

The assay was first evaluated using the boiling method by heating the diluted sample to 95 °C then cooling it to 4 °C followed by the RT-LAMP reaction which showed the lowest LOD of (3.16 pfu/mL) for the S-123 primer set and the combination primer set (S-123/N). In order to reduce the reaction time further, we evaluated the assay at different reaction times (60, 45, 40, 35, and 30 min) with the shortest time of 45 min showing similar LOD to the original time recommended by the manufacturer.

To reduce the reaction time further, we evaluated omitting the boiling step, which consumed 15 min of the assay time. The direct (no boiling) assay was found to have a LOD similar to the boiling assay (3.16 pfu/mL) using the S-123 primer set both alone or in combination with the N primer set.

A second RT-LAMP amplification mix (MMix-B) from New England Biolabs was also evaluated. This master mix showed better performance than MMix-A, where it had the same LOD as MMix-A but at a shorter time (30 min). When tested at 45 min, similar to MMiX-A, MMix-B showed higher LOD (3.16 × 10^−1^ pfu/mL).

The clinical evaluation of the assay was performed on 200 clinical samples and compared to the standardized real-time RT-PCR assay. The assay showed 100% concordance with the real-time assay when performed using MMix-A with a reaction time of 45 min and when tested using MMix-B with a reaction time of 30 min. MMix-B gave a better privilege for the assay in reducing the reaction time to 30 min.

In this study, the cross-reactivity evaluation showed no cross-reactivity with the available MERS-CoV in our facility. The in-silico evaluation of the cross-reactivity of the primers was evaluated previously in [26,27], where they showed no cross-reactivity with other human coronaviruses by aligning the primers with the targeting site in these viruses. Earlier, we used the RT-LAMP test to screen for MERS-CoV [20], and more recently, it was used by others to screen for SARS-CoV-2. Haq and colleagues compared the performance of the colorimetric RT-LAMP assay to the conventional RT-PCR method to detect SARS-CoV-2. The three genes (ORF1ab, N, and S) were identified as target sites for COVID-19 detection in the RT-LAMP assay using extracted RNA samples detected at 65 °C for 60 min. Using the RT-LAMP approach, overall sensitivity of 91.45% and a specificity of 90% were detected [28]. Yu et al. used a colorimetric RT-LAMP assay for the detection of SARS-CoV-2 using ORF1ab gene primers and extracted RNA samples detected at 65 °C for 20 min [29].

Taken together, the results from this study provide a reliable, sensitive, specific, and short turnaround RT-LAMP assay for the detection of SARS-CoV-2 in clinical samples. The use of RT-LAMP technology offers a fast amplification alternative—together with omitting RNA extraction and sample boiling, it resulted in a turnaround time of 30 min. The assay can be used as a point of care testing for the diagnosis of SARS-CoV-2 near or at the site of patient care facilities, transportation hubs, and field studies. The assay is also valuable for SARS-CoV-2 diagnosis in resource-limited areas as it provides a cost-effective and time-saving alternative after omitting the RNA extraction step. Although most of the variants of concern (VOC) are located in the S-gene, the ability of the assay to detect new variants needs to be evaluated with isolates of the known VOC. The primers targeting the N-gene are expected to be able to detect the viremia of specimens from VOC infected cases as this genomic region is more genetically stable than the S-gene.

## 5. Conclusions

Based on the worldwide burden of COVID-19 and the evolving of new variants that might have the potential to increase the spread of the virus, the accessibility of kits and reagents for the detection of SARS-CoV-2 might come to scarcity in developing and underdeveloped countries and resource-limited facilities. In this study, the detection of SARS-CoV-2 utilizing a combination of two target genes (N, and S) considerably improved the LAMP test detection limit according to the recommendation of the WHO. The RT-LAMP assay utilizes the fluorescent-based detection of the amplified product which facilitates the automatic calling of the results and helps reduce potential human error when using color-based amplification assays. Although the main objective of this study is to reduce the cost and running time of the assay for virus detection, for this assay to be utilized at or near patient point of care, the performance of the assay needs to be evaluated on a portable fluorimeter.

## Data Availability

All relevant data for this study is available within this article.
